# Development of an Analytical Method Based on Temperature Controlled Solid-Liquid Extraction Using an Ionic Liquid as Solid Solvent

**DOI:** 10.3390/molecules201219842

**Published:** 2015-12-10

**Authors:** Zhongwei Pan, Zhengquan Wang, Linna Zhu, Zhiming Zhu, Jinying Cai, Xiaoman Shen, Tingli Fan, Yingnan Zhang, Zhixiu Chen

**Affiliations:** 1 School of Chemical and Biological Science, Quanzhou Normal University, Quanzhou, 362000, China; 18065318102@163.com (L.Z.); zhuzhiming1208@sina.com (Z.Z.); 18065318218@163.com (J.C.); qsebike5@pcqs-china.com (X.S.); ftl1226@sina.com (T.F.); 18759966957@163.com (Y.Z.); zhixiu.chen@outlook.com (Z.C.); 2 Hengxing Energy Saving Technology Company Limited of Quanzhou, Fujian 362000, China; Zhenquan2811@sohu.com

**Keywords:** determination of Fe^2+^, tea, temperature controlling solid-liquid separation, ionic liquid as solid solvent

## Abstract

At the present paper, an analytical method based on temperature controlled solid-liquid extraction (TC-SLE) utilizing a synthesized ionic liquid, (*N*-butylpyridinium hexafluorophosphate, [BPy]PF_6_), as solid solvent and phenanthroline (PT) as an extractant was developed to determine micro levels of Fe^2+^ in tea by PT spectrophotometry. TC-SLE was carried out in two continuous steps: Fe^2+^ can be completely extracted by PT-[BPy]PF_6_ or back-extracted at 80 °C and the two phases were separated automatically by cooling to room temperature. Fe^2+^, after back-extraction, needs 2 mol/L HNO_3_ as stripping agent and the whole process was determined by PT spectrophotometry at room temperature. The extracted species was neutral Fe(PT)*_m_*Cl_2_ (*m* = 1) according to slope analysis in the Fe^2+^-[BPy]PF_6_-PT TC-SLE system. The calibration curve was Y = 0.20856X − 0.000775 (correlation coefficient = 0.99991). The linear calibration range was 0.10–4.50 μg/mL and the limit of detection for Fe^2+^ is 7.0 × 10^−2^ μg/mL. In this method, the contents of Fe^2+^ in Tieguanyin tea were determined with RSDs (*n* = 5) 3.05% and recoveries in range of 90.6%–108.6%.

## 1. Introduction

Drinking tea can prevent and cure angiocardiopathy, because it contains many effective ingredients like tea polyphenols, pigments, and polysaccharides and microelements, including iron, copper, and zinc, *etc.* In the human body, iron is a constituent of hemoglobin, myoglobin, the cytochrome system and a variety of enzymes in the blood. Absence of iron could lead to hypoferric anemia and hyperlipemia [1]. Spectrophotometric determination methods, including extraction spectrophotometric determination [2], catalytic atomic-absorption spectrometry [3], flow-injection spectrometry [4] and derivative spectrometry [5] *etc.* are the primary methods of iron analysis. These methods are committed to improving the sensitivity and selectivity of iron determination, without caring about the environmental impact of chemicals used, because of the small amounts of reagent used in the analyses. Modern analytical chemistry is not only concerned with the sensitivity and selectivity of the analytical methods, but also places great importance on the impact of chemicals on the environment and humans despite the very small throughput. Therefore green extraction, determination and regulation of iron content in tea could mean a lot to human health. Ionic liquids (ILs) are generally considered green solvents due to their high thermal stability, very low flammability and negligible vapor pressure, but in particular, for their highly tunable nature, which makes ionic liquids the only truly designer solvents [6,7,8], and so far ILs have drawn lots of attention as novel solvents [9,10,11,12,13]. These characteristics confer them with outstanding properties when they are used as solvents compared with conventional molecular liquids. However some syntheses of ILs in fact, do not involve green processes. Besides, there is no word yet on the water pollution caused by ILs and the natural degradation products of ILs. Many researchers have reported the solvent extraction behavior of metal ions with water immiscible ionic liquids. Hydrophobic ILs with imidazolium cations are frequently used as solvents or diluents in solvent extraction systems [14,15,16,17,18].

Temperature controlled solid-liquid extraction (TC-SLE) is considered a branch of extraction chemistry [19,20]. In this technique, organic phases such as naphthalene, biphenyl, paraffin waxes and *N*-butylpyridinium hexafluorophosphate ([BPy]PF_6_) that are solids at room temperature are employed as extraction solvents when the temperature is higher than their melting points. Water insoluble complexes are readily extracted into the molten organic phases and distribution equilibrium is rapidly achieved at the higher temperature. Phase separation can be easily obtained by cooling the extraction system to room temperature. Some metals generate metal chelates with organic extractants at elevated temperature (for example, acetylacetone chelate [21,22]), so these metals is not suitable for liquid-liquid extraction (LLE). In this case, metal complexes must first be formed at high temperatures, cooled to room temperature and then extracted into a suitable organic solvent. In this way, not only excessive organic solvents are used, but the amount of organic solvent and duration of the separation process are also increased. Thus, based on the synthesis of [BPy]PF_6_, a novel and satisfactory TC-SLE system for the separation and determination of Fe^2+^ in tea was developed with phenanthroline (PT) as an extractant and [BPy]PF_6_ as a “green” solvent.

## 2. Results and Discussion

### 2.1. Optimization of TC-SLE of Fe*^2+^* with [BPy]PF*_6_*-PT

Different factors affect the TC-SLE process. It is very important to optimize them to obtain the best recovery. When the extraction temperature is higher than the melting point of [BPy]PF_6_, the extraction efficiency is not influenced by the temperature. TC-SLE can be achieved within 10 min. As a result, in the experiment a 10 min extraction is performed at 80 °C. The highest extraction efficiency was obtained with 0.5 g~3.0 g of [BPy]PF_6_-PT when the other conditions were kept unchanged. Thus 1 g of [BPy]PF_6_-PT was used in the experiment.

### 2.2. Effect of pH on TC-SLE of Fe*^2+^*

**Figure 1:**
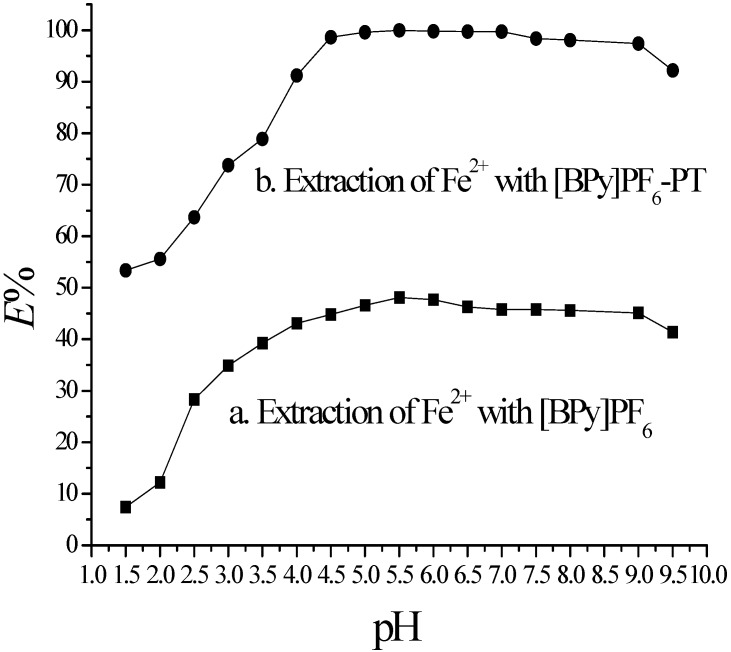
Plot of %E as a function of pH in TC-SLE system. Conditions: IL phase, 1 g [BPy]PF_6_, [PT]_IL_ = 8.10 × 10^−3^ mol·L^−1^; Aqueous phase, [Fe^2+^] = 1.0 × 10^−5^ mol·L^−1^.

The percentage of Fe^2+^ extraction into [BPy]PF_6_ with or without PT as a function of the pH value of the aqueous phase is plotted in Figure 1.

It shows that the Fe^2+^ extraction efficiency is less than 50% at pH 1.50~9.50 when there is no PT in [BPy]PF_6_, and extraction efficiency of Fe^2+^ is higher than 99.6% at pH 5.00~7.00 when there is 8.1 × 10^−3^ mol·L^−1^ PT in [BPy]PF_6_. These results indicate that the optimal pH value was 5.50 for Fe^2+^ extraction at this TC-SLE system. The Fe^2+^ extracted into the solid [BPy]PF_6_-PT phase can be quantitatively back-extracted into 2 mol·L^−1^ HNO_3_ solution at 80 °C. Extraction of Na^+^ or K^+^ into [BPy]PF_6_-PT was negligible.

### 2.3. Effect of PT Concentration on TC-SLE of Fe*^2+^*

**Figure 2:**
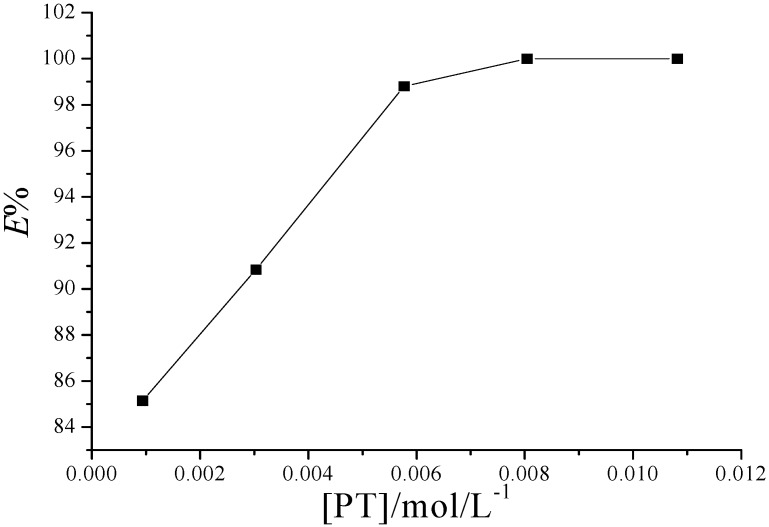
Plot of *%E* as a function of the concentration of PT in TC-SLE system. Conditions: IL phase, 1 g [BPy]PF_6_; Aqueous phase, [Fe^2+^] = 1.0 × 10^−5^ mol·L^−1^, pH = 5.50.

Figure 2 shows the effect of PT concentration in the range of 9.41 × 10^−4^~1.08 × 10^−2^ mol·L^−1^ on Fe^2+^ TC-SLE behavior. When the PT concentration was 8.04 × 10^−3^ mol·L^−1^, the extraction efficiency of Fe^2+^ reached 100% and remained constant at pH = 5.50. Consequently 8.10 × 10^−3^ mol·L^−1^ of PT in [BPy]PF_6_ was selected for determination of Fe^2+^ in tea.

### 2.4. Composition of the TC-SLE Extracted Species

PT can form 1:3 complexes with iron that are suitable for determination of Fe^2+^ [23]. In the presence of large amounts of Cl^−^, the extraction reaction of PT with Fe^2+^ in TC-SLE system can be expressed in the following manner:

(1) $${\text{Fe}}^{2+}+m{\text{(PT)}}_{\text{(IL)}}+2{\text{Cl}}^{\text{-}}\Leftrightarrow {\text{Fe(PT)}}_{m}{\text{Cl}}_{\text{2(IL)}}$$

The extraction equilibrium constant (*K_ex_*) of TC-SLE for Fe^2+^ can be written as follows:

(2) $$K_{ex}=\frac{{{\text{[Fe(PT)}}_{m}{\text{Cl}}_{\text{2}}\text{]}}_{\text{IL}}}{{\text{[Fe}}^{\text{2}+}{\text{][PT]}}_{\text{IL}}^{m}{{\text{[Cl}}^{-}\text{]}}^{\text{2}}}$$

Corresponding *D* of Fe^2+^ between two phases can be expressed as follows:

(3) $$D=\frac{{{\text{[Fe(PT)}}_{m}{\text{Cl}}_{\text{2}}]}_{\text{IL}}}{\left[{\text{Fe}}^{\text{2}+}\right]}=K_{ex}{\left[\text{PT}\right]}_{\text{IL}}^{m}{\left[{\text{Cl}}^{-}\right]}^{2}$$

where [Fe^2+^] and [Cl^−^] are the equilibrium concentration of Fe^2+^ and Cl^−^ in the aqueous phase and [Cl^−^] is regarded as constant as a result of the large amount of Cl^−^ in the TC-SLE system. [PT]_IL_ and [Fe(PT)*_m_*Cl_2_]_IL_ are the equilibrium concentrations of PT and Fe(PT)*_m_*Cl_2_ in the IL phase at 80 °C, and *m*, the composition ratio of PT to Fe^2+^ in the extractive compound.

When Equation (3) is expressed in logarithmic format, it can be rewritten as:

(4) $$\log D=\log K_{ex}+2\log[{\text{Cl}}^{-}]+m\log{\left[\text{PT}\right]}_{\text{IL}}$$

**Figure 3:**
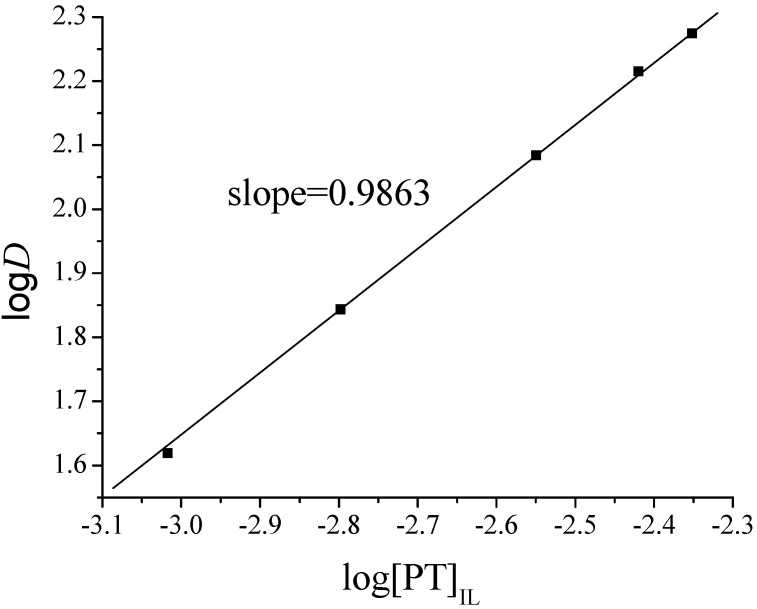
Plot of log*D* as a function of log[PT] in TC-SLE system. pH = 5.50, [Fe^2+^] = 1.0 × 10^−5^ mol·L^−1^. The slope of lines indicated was obtained by the least squares fitting in the figure.

Obviously, log *D* is linear with a slope of *m* along with concentration of PT in IL phase when the temperature and pH are fixed and the concentration of Cl^−^ is kept constant in the TC-SLE system. As shown in Figure 3, the slope of the straight lines was *ca.* 1. The result indicates that Fe^2+^ is extracted into the ionic liquid phase in neutral Fe(PT)*_m_*Cl_2_ (*m* = 1). Thus the extraction mechanism can be expressed by Equation (5):

(5) $${\text{Fe}}^{2+}+{\text{PT}}_{\text{(IL)}}+2{\text{Cl}}^{\text{-}}\Leftrightarrow {\text{Fe(PT)Cl}}_{\text{2(IL)}}$$

### 2.5. Back-Extraction of Fe*^2+^*

**Figure 4:**
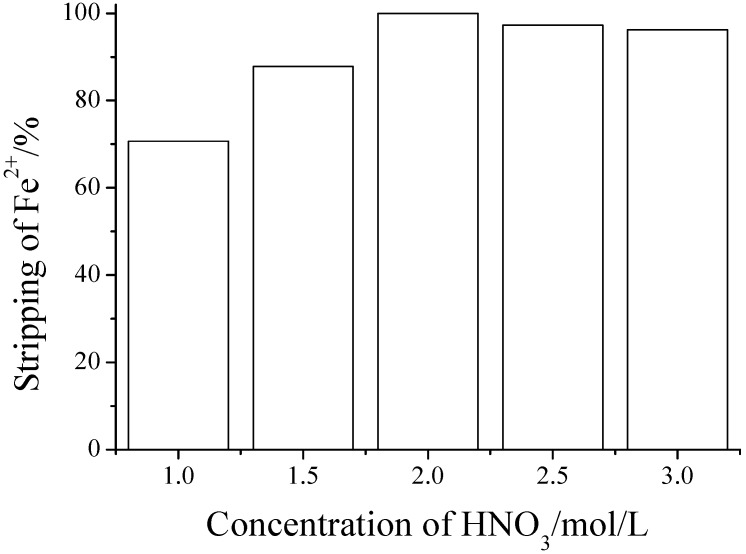
Stripping of Fe^2+^ in TC-SLE system using different concentration of HNO_3_ as stripping solution. IL phase: [PT]_IL_ = 8.10 × 10^−3^ mol·L^−1^; Aqueous phase: initial [Fe^2+^] = 5.5 × 10^−5^ mol·L^−1^.

The experiments show that the effect of back-extraction for Fe^2+^ using HNO_3_ as stripping agent in the TC-SLE system is better. The results are shown in Figure 4, where a 100% Fe^2+^ stripping percentage was obtained using 2 mol·L^−1^ HNO_3_ as stripping agent. Therefore 2 mol·L^−1^ of HNO_3_ was selected as stripping agent in the TC-SLE system for the determination of Fe^2+^ in tea.

After back-extraction of Fe^2+^, the [BPy]PF_6_-PT was washed three times with distilled and deionized water and could be used again in this TC-SLE system. The results indicate that only 8% of [BPy]PF_6_-PT was washed away after triplicate extraction of Fe^2+^ because of the solubility of PT [24] and [BPy]PF_6_ in water as shown by IR. Thus the TC-SLE procedures are simple, rapid, costly effective and environmentally friendly.

### 2.6. Determination of Fe*^2+^* in Tea

Interference tests were conducted with a mixed solution containing 1.25 μg/mL Fe^2+^ and an excess of the possible interfering ions. When the relative error of the absorbancy change was less than ±5%, at least 500 times the amount of K^+^, Na^+^, Mg^2+^, Al^3+^, Ba^2+^, Mn^7+^, Sr^2+^, NH_4_^+^, Cl^−^, I^−^, SO_4_^2−^ and NO_3_^−^ did not interfere with the determination of Fe^2+^, and at least 300 times the amount of Pb^2+^, Cd^2+^, Ga^3+^, In^3+^, Tl^+^, Se^4+^, Hg^2+^, Ag^+^, V^5+^ and Ti^4+^ did not interfere with the determination of Fe^2+^. But a 5-fold excess of Ca^2+^, Fe^3+^ and 10 times the amount of Zn^2+^, Ni^2+^, Mn^2+^ interfered seriously with the determination of Fe^2+^. A mixture of 6% sodium citrate and 0.015 mol·L^−1^ ethylenediamine tetraacetic acid (EDTA) that does not interfere with the Fe^2+^ determination after absorption spectrophotometric testing can effectively mask the interferences of these five cations.

According to the method of this experiment, the regression equation for the determination of Fe^2+^ and its correlation coefficient (R) were: Y = 0.20856X − 0.000775 (R = 0.99991). The calibration graph fitted Beer’s law at a signal-to-noise ratio of 3 (S/N = 3) was found to be 7.0 × 10^−2^ μg/mL. The determination results of Fe^2+^ in Tieguanyin tea after TC-SLE are shown in Table 1. The recovery experiment results for Fe^2+^ in the TC-SLE system are shown in Table 2.

**Table 1:** Determination of Fe^2+^ in Tieguanyin tea.

Sample	Determinate Concentration (μg/mL)	Average Concentration (μg/mL)	RSD	Contents of Fe^2+^/g Dried Tea (μg/g)	Average Contents of Fe^2+^/g Dried Tea(μg/g)	Reference Value [25] (μg/g)
1	1.5294	1.4968	3.05%	609.68	594.68	19.77~797.47
2	1.5161			575.28		
3	1.4481			603.48		
4	1.5429			610.74		
5	1.4475			574.22		

**Table 2:** The recovery rate of the determination of Fe^2+^ in Tieguanyin tea.

Sample	Fe^2+^ Content in the Sample (μg/mL)	Amount of Standard Fe^2+^ Added (μg/mL)	Measured Amount (μg/mL)	Percent Recovery (%)
1	1.4865	0.7430	2.2471	102.4
		1.4885	3.2142	90.6
		2.2255	3.9046	108.6
2	1.5021	0.7430	2.2427	99.7
		1.4885	3.0153	101.7
		2.2255	3.7689	101.9

The experiments were carried out by adding standardized Fe^2+^ into tea samples in accordance with the ratios 0.5:1, 1:1 and 1.5:1. As shown in Table 1, the average Fe^2+^ content per gram of dry Tieguanyin tea was 594.68 μg with a 3.05% of relative standard deviation (RSD) and 90.6%–108.6% recovery for Fe^2+^ as shown in Table 2. The Fe^2+^ determination results reported in this paper are within the range reported in the literature [25]. While references [26,27,28,29] reported similar results as this method for the determination of iron, but the total time required for the determination of iron is longer than in this paper because those methods for the determination of iron include multiple steps such as calcination, dissolution, extraction and back-extraction, *etc.* For the purposes of extraction, the SLE process was completed at 80 °C in this paper and its mass transfer process was faster than LLE [26], solid phase extraction [27], ultrasound assisted-deep eutectic solvent extraction [28] and on-line solid phase extraction [29]. The method presented here is therefore applicable for the quantification of Fe^2+^ with accuracy, precision and reproducibility as listed in Table 1 and Table 2.

## 3. Experimental Section

### 3.1. General Information

PT (0.015%) was freshly prepared prior to use. [BPy]PF_6_-PT (0.02 mol·L^−1^) was prepared by dissolving PT in [BPy]PF_6_ at 80 °C. Stock solution of 1.0 × 10^−2^ mol·L^−1^ Fe^2+^ was prepared using GR NH_4_Fe(SO_4_)_2_·12H_2_O by a usual method. Other chemicals used were of analytical or guaranteed reagent-grade and used without further purification. Distilled water was used throughout unless otherwise specified. An Avatar 360 FT-IR (Nicolet, Waltham, MA, USA) was used to record the IR spectrum. A V-1800 spectrophotometer (Mapada, Shanghai, China) was used for the determination of Fe^2+^. A pHS-3C meter (Jinpeng Analytical Instruments Ltd., Shanghai, China) was employed to monitor the pH values at room temperature. A WRS-1B digital melting point apparatus (Precision & Scientific Instrument Co., Ltd., Shanghai, China) was used to determine the melting point of [BPy]PF_6_. TC-SLE was carried out using the device described in our previous report [30]. In the device, a Model CS501 thermostat (Zhongbao thermostat Co., Ltd., Chongqing, China) was used to control the temperature of extraction vessel with a water jacket and a Model DF-101B magnetic stirrer (Rongkai Industry & Trade Ltd., Luoyang, China) was employed to vigorously stir the solution.

### 3.2. Synthesis and Characterization

**Scheme 1:**
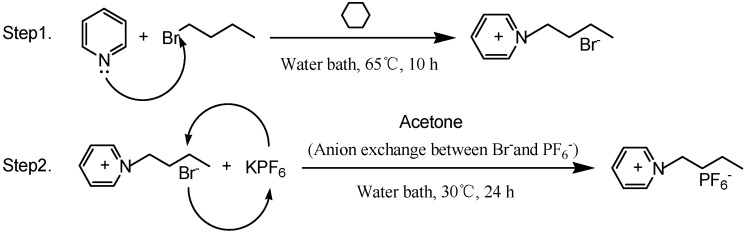
Synthetic steps and structure of [BPy]PF_6_.

The two steps of the synthesis and the structure of [BPy]PF_6_ are shown in Scheme 1 [30].

**Figure 5:**
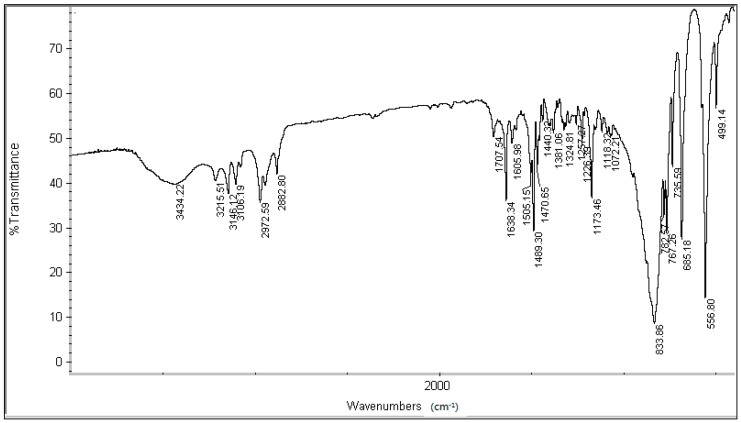
IR of [BPy]PF_6._

The infrared spectrum (IR) of [BPy]PF_6_ (Figure 5) showed the following bands: 3434.22 cm^−1^ corresponds to the -O-H stretching vibration of a small amount of water absorbed in [BPy]PF_6_; 3146.12 cm^−1^ and 3106.19 cm^−1^ are the C-H ring stretching vibrations; 2972.59 cm^−1^ is the asymmetric stretching peak of C-H from -CH_3_; 2882.80 cm^−1^ is the symmetric stretching peak of C-H from -CH_3_; 1638.34 cm^−1^ and 1489.30 cm^−1^ are the stretching vibration of C=N; 1505.15 cm^−1^ and 1470.65 cm^−1^ are characteristic peaks of the pyridine ring; 1440.32 cm^−1^ (med.) is the bending asymmetric vibration peak of -CH_3_; 1173.46 cm^−1^ is the in-plane bending vibration peak of the ring C-H bonds; 782.57 cm^−1^ is the C=C bending vibration peak of the pyridine ring; 685.18 cm^−1^ is the bending (deformation) vibration peak of C-H on the ring and 833.86 cm^−1^ and 556.80 cm^−1^ are characteristic absorption peaks of PF_6_^−^.

The density of [BPy]PF_6_ was determined to be 1.2501 g·mL^−1^ with specific gravity bottle method [31]. The melting point of [BPy]PF_6_ is 73.8 °C~75.5 °C.

### 3.3. Fe*^2+^*TC-SLE Procedure

[BPy]PF_6_ with PT (1 g) and a solution containing the desired amount of Fe^2+^ and buffer (10 mL) were placed in the glass extraction vessel described in [30]. The mixture was stirred on the magnetic stirrer at 80 ± 0.1 °C for 10 min and cooled to room temperature. After the organic phase ([BPy]PF_6_-PT phase) solidified and separated, the acidity of aqueous phase was measured by a pH meter and the amount of Fe^2+^ in the aqueous phase was determined by PT spectrophotometry [24]. The concentration of Fe^2+^ in the [BPy]PF_6_-PT phase was determined after back-extraction at 80 °C for 10 min in 10 mL of 2 mol·L^−1^ HNO_3_. The extraction percentage (*%E*) of Fe^2+^ was calculated by the ratio of the extracted amount of Fe^2+^ to the initial amount of Fe^2+^ in the aqueous phase. The distribution ratio (*D*) was estimated by the ratio of total concentration of Fe^2+^ in the solid phase to that in the aqueous phase. In the experiments, the ionic strength was maintained at 0.1 with sodium chloride.

### 3.4. Tea Sample Preparation

Commercial Tieguanyin tea (1.000 g, pulverized in advance) was accurately weighed, then powdered after drying at 75–80 °C. The tea powder were put in a porcelain crucible and soaked into 0.2 mol·L^−1^ HCl. After that the wetted tea powders were burned in a muffle furnace at 500 °C for 4 h then the wet tea powders were carbonized in an electric cooker at low temperature, and then the porcelain crucible was taken out and cooled to room temperature. 1:1 HCl was added into the porcelain crucible to dissolve the residue, the solution was filtered and the pH was adjusted to 7 or so and then transferred to a 100 mL volumetric flask to stand after adjusting to a constant volume.

## 4. Conclusions

A novel TC-SLE system with an eco-friendly IL as a solid extraction solvent has been developed for the separation and determination of iron. Its extraction behavior is different from that of a liquid-liquid system [32]. The IL phase becomes solid when the temperature decreases to room temperature. After solid-liquid separation, a quantitative extraction of Fe^2+^ is performed. Furthermore the determination of Fe^2+^ at tea can be achieved based on a fixed pH at room temperature. The unique properties of the IL allow a highly efficient and selective separation and determination of iron. Solid ILs can be recycled. Small amounts of water contained in [BPy]PF_6_ as determined by IR, may have contributed to the wide range of recoveries observed (90.6%–108.6%). The proposed TC-SLE with IL provides an alternative route for the separation and determination of iron in different matices.
